# Predictive Biomarkers for Asymptomatic Adults: Opportunities, Risks, and Guidance for General Practice

**DOI:** 10.3390/diagnostics16020196

**Published:** 2026-01-08

**Authors:** Christian J. Wiedermann, Giuliano Piccoliori, Adolf Engl, Doris Hager von Strobele-Prainsack

**Affiliations:** Institute of General Practice and Public Health, Claudiana—College of Health Professions, 39100 Bolzano, Italy

**Keywords:** preventive medicine, biomarkers, polygenic risk scores, direct-to-consumer testing, primary care, overdiagnosis, risk communication, ethical considerations, general practitioners

## Abstract

Biomarker-based prevention is rapidly expanding, driven by advances in molecular diagnostics, genetic profiling, and commercial direct-to-consumer (DTC) testing. General practitioners (GPs) increasingly encounter biomarker results of uncertain relevance, often introduced outside the guideline frameworks. This creates new challenges in interpretation, communication, and equitable resource use in primary care. This narrative review synthesizes evidence from population-based studies, guideline frameworks, consensus statements, and communication research to evaluate the predictive value, limitations, and real-world implications of biomarkers in asymptomatic adults. Attention is given to polygenic risk scores, DTC genetic tests, neurodegenerative and cardiovascular biomarkers, and emerging multi-omics and aging markers. Several biomarkers, including high-sensitivity cardiac troponins, N-terminal pro–B-type natriuretic peptide, lipoprotein(a), coronary artery calcium scoring, and plasma p-tau species, showed robust predictive validity. However, many widely marketed biomarkers lack evidence of clinical utility, offer limited actionable benefits, or perform poorly in primary care populations. Unintended consequences, such as overdiagnosis, false positives, psychological distress, diagnostic cascades, and widening inequities, are well documented. Patients often misinterpret unvalidated biomarker results, whereas DTC testing amplifies demand without providing adequate counseling or follow-up. Only a minority of biomarkers currently meet the thresholds of analytical validity, clinical validity, and clinical utility required for preventive use in general practices. GPs play a critical role in contextualizing biomarker results, guiding shared decision-making, and mitigating potential harm. The responsible integration of biomarkers into preventive medicine requires clear communication, strong ethical safeguards, robust evidence, and system-level support for equitable, patient-centered care.

## 1. Introduction

The expansion of biomarker testing has transformed preventive medicine. Molecular, imaging, and digital biomarkers, from high-sensitivity cardiac troponins and N-terminal pro–B-type natriuretic peptide (NT-proBNP) to plasma p-tau species, polygenic risk scores (PRS), and multi-cancer detection assays, are marketed to asymptomatic adults for personalized risk prediction. Direct-to-consumer (DTC) genetic testing and private screening panels have increased public demand for early detection technologies, often outpacing the evidence for their safe use [1,2,3]. While several biomarkers have shown predictive validity in large prospective studies, few have demonstrated improvements over established clinical risk tools or shown clinical utility in preventing disease [4,5,6,7]. Many emerging tests lack sufficient validation, actionable interventions, and consistent performance across populations [8,9]. Premature adoption can lead to false positives, overdiagnosis, psychological distress, and inequitable access, consequences that are particularly relevant in primary care, where disease prevalence is low and interpretative uncertainty is high [10,11,12].

GPs are frequently asked to interpret commercial biomarker results or facilitate testing, even when the tests fall outside standard clinical indications [13,14]. This creates pressure on consultation time, communication skills, and resource stewardship and underscores the need for evidence-based frameworks to guide decision-making.

This narrative review synthesizes evidence on the predictive value and limitations of biomarkers in preventive medicine, including cardiovascular, metabolic, neurodegenerative, oncological, and genetic markers. It examines PRS, DTC testing reliability, unintended consequences of biomarker screening, communication challenges, ethical considerations and implications for general practice. The aim was to provide primary care clinicians with an evidence-informed perspective on the responsible integration of biomarkers into preventive care while avoiding harm, unnecessary interventions, and widening inequities.

## 2. Methods

This narrative review synthesizes evidence on the predictive value, limitations, and implications of biomarker-based prevention in asymptomatic adults for general practice. Literature was obtained through Consensus (Consensus AI, Inc., Boston, MA, USA), an AI-enabled search engine that indexes and retrieves research papers published online from PubMed, Semantic Scholar, and other scholarly databases, using the Pro version with Deep answers available at the time of the search. Search terms included “preventive biomarkers,” “primary care,” “risk prediction,” “polygenic risk score,” “biomarker communication,” “overdiagnosis,” and “clinical utility.”

Consensus AI (Medical Mode 20 November 2025) was employed to support efficient identification of peer-reviewed studies, guidelines, and consensus statements across multiple databases. As an AI-assisted tool, it may underrepresent very recent or non-indexed sources; therefore, all outputs were critically appraised by the authors. AI-assisted retrieval was complemented by manual searches of reference lists and clinical guidelines to verify and contextualize evidence. Manual searches by the lead author focused on documents from professional organizations (e.g., AHA, ASCO, ESC, NIH, Alzheimer’s Association) providing formal recommendations on preventive biomarker use in asymptomatic adults. Guidelines were selected based on relevance to primary care prevention, clinical utility, and applicability to population-based settings.

The disease domains included cardiovascular, metabolic, oncological, and neurodegenerative diseases. Additional searches covered patient interpretation, communication strategies and GP responses to private biomarker testing.

The literature search was conducted between October and November 2025. No publication date restrictions were applied. The identified literature spans the period from 1996 to 2025, covering foundational methodological work as well as recent advances in biomarker-based preventive medicine.

Evidence synthesis followed integrative narrative analysis principles, focusing on clinical validation, harm, and implementation considerations from a primary care perspective. This narrative review did not involve duplicate independent screening or formal study selection procedures, as it does not aim to achieve systematic completeness. Priority was given to systematic reviews, large cohort studies, randomized trials, and guidelines from professional organizations, as these sources provide the most robust and clinically generalizable evidence for preventive decision-making in asymptomatic populations. Case reports, small exploratory studies, purely technical assay validations, and studies conducted exclusively in symptomatic or secondary-care populations were not prioritized unless they addressed ethical, communicative, or implementation-relevant aspects applicable to primary care. This review emphasizes the risk of overdiagnosis, implications of commercial testing, and communication challenges. The quality of this narrative review was appraised against the domains of the Scale for the Assessment of Narrative Review Articles (SANRA) [15], which is intended to evaluate clarity, transparency, and balance in narrative evidence synthesis. This review does not aim to systematically map the available literature and therefore does not follow other reporting standards developed for scoping or systematic reviews.

Generative artificial intelligence (ChatGPT-5.2, OpenAI, Inc., San Francisco, CA, USA) was used to support the drafting of narrative text, to assist in synthesizing the published literature identified through conventional and AI-assisted searches, and to structure sections of the manuscript based on author-provided content and references. The authors have reviewed and edited the output and take full responsibility for the content of this publication.

## 3. Predictive Biomarkers and Polygenic Risk Scores in Preventive Medicine

Biomarker-based risk prediction in asymptomatic adults has advanced significantly over the past decade. Beyond traditional risk factors, cardiovascular, metabolic, neurodegenerative, and cancer-related biomarkers have shown strong predictive value in large cohorts and meta-analyses. Although their improvement over established scores is modest, biomarkers such as high-sensitivity cardiac troponins, NT-proBNP, high-sensitivity C-reactive protein (hs-CRP), and imaging markers such as coronary artery calcium (CAC) scoring offer gains in discrimination and reclassification. This section synthesizes validated biomarkers with predictive utilities in asymptomatic populations.

### 3.1. Cardiovascular Biomarkers

#### 3.1.1. High-Sensitivity Cardiac Troponins (hs-cTnI, hs-cTnT)

High-sensitivity cardiac troponin levels strongly predict future cardiovascular events in asymptomatic adults. Elevated baseline hs-cTn levels predict myocardial infarction, heart failure, and cardiovascular mortality, independent of conventional risk factors [16,17,18,19,20]. Their integration modestly improved risk model discrimination, particularly in intermediate-risk individuals. Beyond acute coronary syndrome diagnosis, population data now support the role of troponins in long-term risk prediction, indicating subclinical myocardial stress as a preventive signal. In preventive settings, hs-cTn is interpreted as a continuous risk marker at concentrations well below diagnostic cut-offs, independent of assay-specific or sex-specific thresholds used for acute myocardial infarction diagnosis.

#### 3.1.2. N-Terminal Pro–B-Type Natriuretic Peptide (NT-proBNP)

NT-proBNP, a biomarker of cardiac stress, predicts outcomes such as heart failure, atrial fibrillation, and cardiovascular mortality in asymptomatic adults [17,20]. NT-proBNP outperforms traditional predictors and enhances risk assessment in multimarker panels [21]. It identifies subclinical heart disease, enabling early and preventive interventions.

#### 3.1.3. High-Sensitivity C-Reactive Protein (hs-CRP)

hs-CRP reflects systemic low-grade inflammation and predicts cardiovascular events across populations [16,19]. Although its predictive improvement is modest, hs-CRP is valuable for adults with intermediate-risk profiles or discordant inflammatory and lipid markers. This supports the role of vascular inflammation in atherosclerosis and highlights the benefits of integrating inflammatory and cardiac biomarkers.

#### 3.1.4. Lipoprotein(a)

Lipoprotein(a) [Lp(a)] is a genetically determined, causal risk factor for atherosclerotic cardiovascular disease and aortic stenosis, with associations demonstrated across multiple population studies [22,23,24,25,26]. Higher Lp(a) concentrations predict cardiovascular events in diverse cohorts, showing a linear relationship with risk [27,28,29,30,31,32,33,34]. Adding Lp(a) to established risk equations improves prediction, particularly among individuals at intermediate cardiovascular risk [27,28,30,32,33]. Several major organizations now recommend one-time Lp(a) measurement during adulthood to refine risk stratification [22,25,33]. Nevertheless, the discrimination gain is small [27,28], assay standardization remains inconsistent [23,24,25], and evidence that lowering Lp(a) reduces events is still emerging pending trials [22,25,34]. In practice, Lp(a) can help explain residual cardiovascular risk and support LDL-cholesterol–lowering strategies in selected patients. However, it does not function as a standalone predictive biomarker for screening asymptomatic adults and should be interpreted within clinical and familial context.

### 3.2. Imaging Biomarkers

#### 3.2.1. Coronary Artery Calcium (CAC) Scoring

CAC scoring outperformed serum biomarkers in predicting coronary heart disease in asymptomatic populations. Large studies have shown that CAC improves reclassification among adults with borderline or intermediate 10-year cardiovascular risk [35,36,37,38]. CAC integrates lifetime exposure to risk factors and identifies subclinical atherosclerosis more directly than biochemical measurement. Its consistent predictive power has led investigators to consider CAC as the benchmark imaging biomarker for preventive cardiology [39,40].

#### 3.2.2. Carotid Plaque Burden

Carotid ultrasound imaging to evaluate plaque presence and burden can predict future cardiovascular events. Studies comparing carotid and coronary imaging show that both improve risk stratification, with CAC performing better, but carotid plaque provides complementary information [41]. Carotid plaque assessment is useful in cases where CT-based CAC is less accessible.

### 3.3. Metabolic Biomarkers

#### 3.3.1. Metabolomic Signatures for Type 2 Diabetes

Metabolomic profiling in large cohorts has shown that specific amino acids, lipid fractions, and metabolic signatures predict long-term type 2 diabetes risk among young asymptomatic adults [42]. These biomarkers outperform traditional glucose-based measures in early risk detection and highlight the metabolic perturbations that precede hyperglycemia. Although clinical implementation remains limited, evidence supporting their predictive validity continues to grow.

#### 3.3.2. Autoantibodies for Type 1 Diabetes

Autoantibodies targeting pancreatic islet antigens are biomarkers that predict the progression to type 1 diabetes, even years before clinical onset. Their use enables early detection, staging, and preventive intervention in at-risk individuals [43]. Although they are used in high-risk cohorts, evidence supports their high predictive accuracy.

#### 3.3.3. Proteomic Panels for Diabetic Kidney Disease

Proteomic risk panels, such as PromarkerD, can identify asymptomatic individuals with type 2 diabetes at high risk of future diabetic kidney disease, preceding albuminuria, or eGFR decline [44]. These markers may significantly improve renal risk assessment in patients with early metabolic dysfunction.

### 3.4. Neurodegenerative Biomarkers

#### 3.4.1. Plasma p-Tau Species (p-Tau181, p-Tau217)

Plasma phosphorylated tau biomarkers are highly predictive of early Alzheimer’s disease (AD) pathology. Studies show p-Tau217 and p-Tau181 predict amyloid accumulation and positron emission tomography (PET)-confirmed preclinical AD with an accuracy comparable to that of neuroimaging [45,46]. These biomarkers reflect neuropathological processes before the onset of clinical symptoms, making them candidates for population-level risk stratification.

#### 3.4.2. Imaging Biomarkers (Amyloid PET)

Amyloid PET imaging can predict the conversion from preclinical to symptomatic AD. Models combining PET with plasma biomarkers enhance their predictive performance [47,48]. Although limited by cost and accessibility, these findings demonstrate the potential of multimodal prediction strategies.

#### 3.4.3. Genetic Risk Markers

Apolipoprotein E (APOE) genotyping is not recommended for AD prevention in general practice. Although APOE ε4 is the strongest genetic risk factor for late-onset AD, its clinical utility is limited. Guidelines discourage APOE testing outside research or therapeutic contexts because of its modest predictive value [49,50,51,52]. The ε4 allele increases AD risk but lacks discriminative power: many ε4 carriers never develop AD, while many non-carriers do [53,54]. Lifestyle interventions for cognitive health are recommended, regardless of genotype [55,56,57]. The main clinical use of APOE genotyping is risk stratification for amyloid-related imaging abnormalities before anti-amyloid therapies, as ε4 carriers require modified treatment approaches [58,59,60]. Outside this context, APOE testing remains limited to research trials [61,62]. While disclosure may motivate health behavior changes, studies show psychological risks, including anxiety [51,63,64]. Insurance and employment implications further complicate this implementation. The role of APOE genotyping remains confined to therapy selection and research, pending advances in personalized interventions.

### 3.5. Non-Invasive Biomarkers for Cancer Detection and Biological Aging

#### 3.5.1. Urinary Glycosaminoglycan Profiling in Elevated Cancer Risk

Urinary glycosaminoglycan (GAGome) profiling identifies individuals with elevated multi-cancer risk beyond traditional screening. Recent studies have shown that abnormal GAGome signatures can predict cancers in asymptomatic adults [65,66]. Although requiring validation, this represents a promising avenue for noninvasive multi-cancer risk assessment. However, improved detection rates or shifts toward earlier cancer stages do not necessarily translate into reduced cancer-specific mortality, and reliance on stage-based surrogate end points may substantially overestimate clinical benefit.

#### 3.5.2. Epigenetic and Biological Age Biomarkers

Epigenetic clocks and biological age algorithms derived from methylation markers predict mortality, hospitalization, and chronic disease risk across large population datasets [67,68,69]. Their specificity for individual diseases remains limited; however, their predictive association with multisystem aging processes makes them potential screening tools for general health-risk assessment.

### 3.6. Polygenic Risk Scores

Polygenic risk scores (PRSs) are tools that quantify inherited susceptibility to complex diseases by combining the additive effects of common genetic variants across the genome [70,71]. Derived from genome-wide association studies, PRSs weigh risk alleles by estimated effect size and sum these into a continuous measure of genetic risk. Unlike monogenic markers, PRSs capture the polygenic architecture and provide probabilistic disease risk estimates that can be compared across individuals, often expressed as population percentiles [70,71].

#### 3.6.1. Predictive Potential and Emerging Clinical Applications

PRSs aggregate the effects of genetic variants to quantify disease susceptibility. The strongest evidence base lies in cardiovascular disease, certain cancers, and type 2 diabetes [72,73,74,75]. PRSs independently predict risk and enhance risk stratification beyond that of clinical models. For coronary artery disease, individuals with a high PRS show an elevated lifetime risk, even without elevated LDL-C or hypertension [72,75,76]. This enables the use of PRSs to guide statin initiation and prevention strategies [77,78,79]. In oncology, breast and prostate cancer PRSs can be used to identify candidates for intensified screening. Models combining PRS, family history, and clinical factors have outperformed traditional tools [74,80,81]. For type 2 diabetes, PRSs predict the risk before the appearance of metabolic abnormalities [76,82]. While predictive gains are modest, PRSs can refine risk estimates and improve the cost-effectiveness of prevention models [83,84].

#### 3.6.2. Limitations, Risk of Bias, and Ethical Considerations

Despite their potential, PRSs face key limitations in primary care. Their modest improvement over clinical risk models questions their value in asymptomatic individuals [85,86]. Current PRSs perform poorly in non-European populations, as genome-wide association studies primarily use European cohorts, leading to the misclassification of underrepresented groups [87,88,89]. This may worsen health disparities and requires more diverse genomic datasets [90,91]. The implementation of PRS also poses ethical challenges. While the psychological impact is minimal, some individuals experience anxiety [92,93,94]. Privacy, discrimination, and consent issues remain problematic [95,96]. Limited provider knowledge and the lack of standardized frameworks hinder adoption [97,98].

#### 3.6.3. Implementation and Future Directions

While modeling studies suggest that PRS-guided prevention may improve the cost-effectiveness of targeted screening, the evidence remains theoretical, with limited real-world validation [79,83,99]. Implementation barriers include electronic health record integration, provider training, and equitable access [94,97]. Future priorities include improving PRS performance across populations, evaluating outcomes, and developing ethical frameworks for privacy and consent issues. Stakeholder-informed implementation and ethical governance are crucial for maintaining equity and patient autonomy [90,91,96]. While promising for personalized prevention, the current limitations of polygenic risk scores mean that they remain emerging rather than established preventive care tools.

### 3.7. Reliability of Direct-to-Consumer Biomarker Tests

DTC genetic tests for cardiovascular and cancer risks show limited reliability. While single nucleotide polymorphism (SNP)-chip-based DTC platforms show acceptable analytic validity for common variants, their clinical validity in predicting disease risk remains modest for conditions such as coronary artery disease and cancers [100,101,102]. Studies have revealed high false-positive rates for rare variants, such as BRCA1/2 and cardiomyopathy mutations, with up to 40% failing clinical confirmation [103,104,105]. DTC companies’ simplified PRS explains minimal disease variance beyond clinical risk factors [77,95,106,107]. Risk estimates vary significantly between companies analyzing identical genetic data because of differences in methods and interpretation [100,102]. Consumers often misunderstand genetic risk, and negative results may provide false reassurance [104,108]. Third-party interpretation services frequently overcall pathogenic variants [102,104]. DTC risk results show minimal impact on sustained lifestyle changes, whereas some users experience increased anxiety [100,108]. Professional societies emphasize that DTC genetic findings require clinical confirmation and counseling [101,103]. While these tests may increase awareness, their use in preventive care requires careful interpretation in clinical settings.

### 3.8. Synthesis

Evidence from cardiovascular, metabolic, neurodegenerative, and oncological research shows that several biomarkers—most consistently hs-cTn, NT-proBNP, hs-CRP, CAC scoring, and plasma p-Tau species—provide validated predictive information in asymptomatic adults. Although their incremental value over established risk factors remains modest, their complementary biological domains suggest that multimodal biomarker combinations may ultimately provide the most informative frameworks for personalized preventive strategies.

Beyond these better-validated biomarkers, a second group of markers including carotid plaque burden, metabolomic signatures for early type 2 diabetes and proteomic panels for diabetic kidney disease, show reproducible associations with future disease risk but offer limited actionable pathways in general practice. Their clinical utility remains constrained by uncertain thresholds, evolving standardization, and the absence of clearly defined interventions.

A third group of early-stage biomarkers, such as PRS, epigenetic clocks and biological age algorithms, GAGome, and broad multi-cancer early detection tests, demonstrate promising research signals but currently lack sufficient validation for preventive use. Their real-world impact is uncertain, and their introduction into primary care without robust evidence may increase inequity, misclassification, and overdiagnosis.

Finally, DTC testing, particularly SNP-chip rare-variant screening, DTC-PRS interpretations, and commercial “biological age” or wellness panels, frequently generates misleading results, false reassurance, or unnecessary anxiety. These tests currently provide little clinical value in asymptomatic adults and commonly lead to diagnostic cascades, increased consultation burden, and patient confusion.

**Figure 1:**
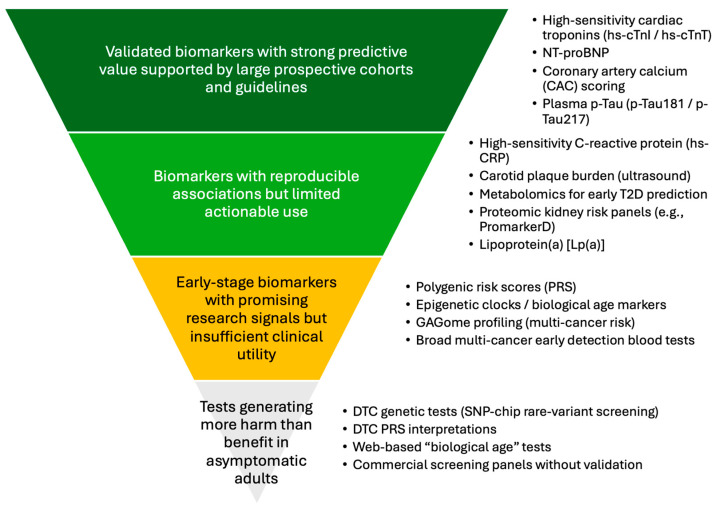
Evidence Hierarchy of Predictive Biomarkers for Asymptomatic Adults. The pyramid emphasizes that only a small subset of biomarkers currently meets the thresholds of analytical validity, clinical validity, and clinical utility required for use in preventive general practice. This hierarchy is a conceptual framework based on qualitative synthesis of the evidence and does not represent formal evidence grading or a GRADE-based assessment. Abbreviations: hs-cTnI/hs-cTnT, high-sensitivity cardiac troponin I/T; NT-proBNP, N-terminal pro–B-type natriuretic peptide; CAC, coronary artery calcium; p-Tau, plasma phosphorylated tau; hs-CRP, high-sensitivity C-reactive protein; Lp(a), Lipoprotein(a); T2D, type 2 diabetes; PRS, polygenic risk score; GAGome, glycosaminoglycan profile; DTC, direct-to-consumer; SNP, single-nucleotide polymorphism.

Together, these tiers illustrate the wide variability in predictive accuracy, actionability, and real-world utility across biomarker categories. Figure 1 summarizes this hierarchy and highlights where meaningful evidence exists for preventive use in general practice.

## 4. Patient Interpretation of Unvalidated Biomarker Results

As biomarker testing expands into commercial and preventive health markets, patients face results that lack clinical utility. While patients generally trust and recall test results, misunderstandings and emotional distress occur when results are unvalidated or poorly contextualized, creating challenges for GPs who interpret commercial findings. Studies have shown that over 80% of patients accurately recall their biomarker results and view them as reliable [109]. This trust, independent of clinical validity, leads patients to consider unvalidated biomarkers as authoritative health information. Confusion is higher among those with lower health literacy who struggle with risk interpretation [109,110]. While patients acknowledge lifestyle and environmental factors in health outcomes [109], some may overvalue biomarker signals without guidance. Unvalidated results can cause mixed emotions, reassurance when favorable, but anxiety when findings are ambiguous or lack explanation [109,111], especially regarding cancer or genetic predispositions.

Only 1% of patients discuss unvalidated biomarker findings with their healthcare providers [109]. Instead, they seek advice from online forums and social media, increasing the risk of misinterpretation. Patients often modify their health behaviors based on these findings, adopting unproven interventions or avoiding necessary clinical evaluations [110,111]. When seeking interpretation help, many turn to online resources [111]. While these communities provide emotional support, they can spread misinformation and oversimplified risk narratives, reinforcing misunderstandings regarding unvalidated biomarkers.

The way patients interpret unvalidated biomarker results impacts primary care in the following ways: misinterpretation persists as patients rarely consult GPs; health anxiety or false reassurance affects preventive behavior; unvalidated results generate additional consultations once patients seek clarification; and GPs must provide retrospective counseling, complicating interpretation.

This highlights the need for structured communication strategies and patient education regarding the limitations of biomarker testing.

Patients trust unvalidated biomarker results but often misunderstand them, leading to anxiety or reliance on non-professional interpretations. Few patients consult healthcare providers, risking inappropriate responses. GPs must contextualize their findings and communicate them clearly to reduce potential harm.

## 5. Harms and Ethical Challenges in Preventive Biomarker Use

Biomarker-based strategies can produce meaningful preventive insights but also introduce several well-documented harms when applied to asymptomatic adults. The most frequent consequences include false positives with downstream diagnostic cascades, overdiagnosis of indolent conditions, psychological distress from ambiguous results, and widening inequities due to uneven access to follow-up care. These risks are amplified when biomarkers lack validated thresholds or actionable treatment pathways.

### 5.1. Overdiagnosis, Overtreatment, and Diagnostic Cascades

Overdiagnosis—detecting abnormalities that would never cause symptoms or affect lifespan—remains a central risk of biomarker-driven screening. This is well documented in cancer screening, where biomarkers for breast, prostate, thyroid, and lung cancers identify large numbers of indolent lesions without reducing mortality [112,113,114,115,116,117,118,119,120,121]. When biomarker thresholds developed in specialist settings are applied in primary care, benign findings may be labeled as disease, prompting unnecessary interventions, follow-up procedures, and avoidable psychological and economic burdens [112,116,117,120].

Because disease prevalence is low in primary care, false-positive results are common and frequently trigger imaging, laboratory testing, and referrals that do not improve outcomes [113,114,122,123,124,125,126,127,128,129,130]. Illustrative examples include unnecessary liver imaging following biomarker panels [122], PSA-initiated cascades of low-yield testing [117], and extensive work-ups after Alzheimer’s biomarker signals [124,126]. Beyond individual patients, such cascades strain practice workflows: they increase coordination needs, amplify testing demands, and shift attention away from established preventive activities [9,124,125,131,132]. The resulting “diagnostic downshift”—the transfer of specialist-oriented testing into primary care—further increases ambiguous findings and complicates decision-making [125]. As broader biomarker panels become commercially available, these system-level pressures are expected to intensify in the absence of clear evidence-based pathways.

### 5.2. Psychological Distress and Uncertainty

Biomarker screening can impose substantial psychological burdens. Anxiety, distress, worry about the disease, and feeling labeled as “at risk” are well described in the screening domains [113,129,130,133,134]. Women receiving abnormal HPV primary screening results often experience anxiety, even when follow-up tests prove normal [130]. Patients undergoing biomarker-based liver or cancer screening may experience psychosocial consequences even without a confirmed diagnosis [129].

### 5.3. Stigma, Discrimination, and Privacy

The expansion of biomarker testing in preventive settings raises ethical challenges when offered outside clinical indications. While patients often view biomarker risk information as empowering, unvalidated testing can cause psychological distress and strain decision making. Ethical evaluation is essential to ensure that biomarker use promotes patient welfare and autonomy.

Predictive biomarker testing, especially for neurodegenerative conditions, can generate psychological burdens. Studies have shown increased anxiety in individuals receiving elevated-risk results, even without effective preventive interventions [11,135,136]. Uncertain findings and false positives heighten distress and prompt unnecessary procedures [10,137].

Expanding testing to asymptomatic populations can identify abnormalities that are unlikely to cause symptoms, leading to medicalization and unnecessary treatment [10,138]. This shifts disease definitions and can be amplified by commercial incentives for broad screening panels [139].

Patient autonomy requires an understanding of the limitations and uncertainties of preventive biomarkers. Informed consent is complex because of long-term uncertain risks, incidental findings, and potential lack of interventions [10,11,135]. Ethical guidelines recommend offering biomarkers only when the findings are clinically actionable with effective interventions [137]. For biomarkers with limited actionability, such as Alzheimer’s or psychiatric risks, testing may burden patients with unusable information.

Preventive biomarker results can affect social dynamics. Risk disclosure for conditions such as dementia may lead to stigma and discrimination [1,140,141]. Privacy concerns are critical for genomic and neurodegenerative biomarkers, affecting families across generations and hindering research. Biomarker testing outside standard indications may increase healthcare disparities through limited access and follow-up care [11,142]. Resources directed toward unproven screening could be diverted from proven public health interventions [10,137].

### 5.4. Equity, Justice, and Societal Implications

Private biomarker testing creates equity challenges, with patients of higher socioeconomic status having greater access, while others rely on limited public systems [3]. GPs should support equitable access to public pathways. Excessive private testing can divert attention from validated preventive interventions. GP advocacy can help promote responsible biomarker use and improve the availability of public testing [14,143]. Biomarker studies often lack representation of disadvantaged groups and those with multimorbidity [124,131,132,134]. Differences in health literacy and access to follow-up care can worsen these disparities. Direct-to-consumer tests target affluent individuals, while underserved groups may miss validated screening but face risks from misinterpreted commercial testing results.

**Figure 2:**
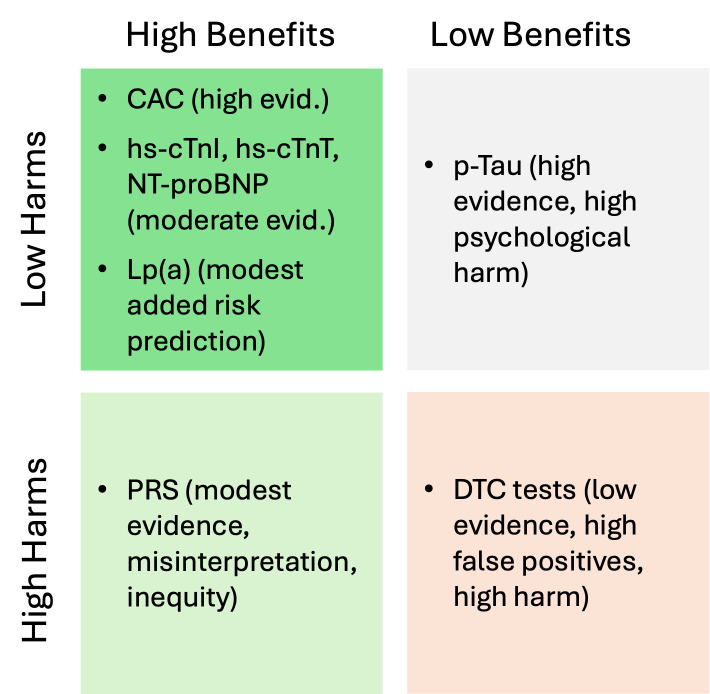
Risk–benefit matrix for biomarker testing in asymptomatic adults. This matrix contrasts the relative benefits and harms of selected biomarkers used or promoted for preventive purposes. Abbreviations: CAC, coronary artery calcium; evid., evidence; hs-cTnI/hs-cTnT, high-sensitivity cardiac troponin I/T; NT-proBNP, N-terminal pro–B-type natriuretic peptide; Lp(a), lipoprotein(a); p-Tau, plasma phosphorylated tau; PRS, polygenic risk score; DTC, direct-to-consumer .

Evidence shows that biomarker-based screening in primary care can have significant unintended consequences. As availability increases, primary care must balance the detection benefits against the risks of overdiagnosis, unnecessary interventions, and inequality. Integration, education, and stronger clinical utility evidence are essential for minimizing these effects. To contextualize these unintended consequences, it is helpful to compare the relative benefits and harms of the most discussed biomarkers in preventive medicine. Although several tests provide meaningful predictive value, others offer limited clinical utility or carry disproportionate risks when applied to asymptomatic adults. A simplified risk–benefit matrix highlights how these biomarkers differ in evidence strength, actionability, and potential for harm, underscoring why careful evaluation is essential before integrating them into routine general practice (Figure 2).

## 6. Frameworks and Guidelines for Evaluating Biomarker Usefulness in Preventive Medicine

Evaluating the contribution of biomarkers to preventive care requires evidence-based frameworks. Major organizations, including the U.S. National Institutes of Health (NIH), American Heart Association (AHA), American Society of Clinical Oncology (ASCO), National Academy of Clinical Biochemistry (NACB), and European guideline groups have developed criteria for assessing analytical validity, clinical validity, and clinical utility [4,5,6,9,144,145]. These frameworks ensure that biomarkers in preventive care improve outcomes, avoid harm, and remain feasible and equitable in practice.

### 6.1. General Evaluation Frameworks

The phased biomarker development framework by Pepe et al. [144] outlines the progression from discovery through validation to clinical utility proof and has been adapted across diseases and NIH programs. Key criteria include analytical validity, measurement accuracy, and reproducibility [4,146]; clinical validity, disease risk associations established through prospective studies [8,147]; and clinical utility, evidence of improved outcomes through biomarker-guided management [6,148,149]. Health technology assessment (HTA) methods evaluate the feasibility and cost-effectiveness of primary care implementation [9,150]. For most preventive biomarkers in asymptomatic populations, robust and generalizable cost-effectiveness estimates are lacking or rely on model-based assumptions, limiting the applicability of incremental cost-effectiveness ratios to routine primary care.

Clinical domains have developed structured biomarker evaluation frameworks.

• Cardiovascular disease: The AHA and NACB guidelines require biomarkers to provide value beyond risk scores and validation in prospective studies [4,5,135]. High-sensitivity cardiac troponins and NT-proBNP meet these criteria.
• Oncology: The ASCO, College of American Pathologists (CAP), and European Group on Tumor Markers (EGTM) guidelines require analytical validation and evidence of benefit [6,7,136,148]. Cervical cancer screening frameworks [8] can serve as templates.
• Neurology: The Alzheimer’s guidelines specify standards for validation and reporting [137].
• Nephrology: Kidney injury and sepsis guidelines integrate clinical utility considerations [138,139].

Guidelines consistently require validated evidence before incorporating biomarkers into the preventive pathways.

### 6.2. Omics, Machine Learning, and Equity

As multi-omic biomarkers and machine-learning-derived risk models enter preventive medicine, new methodological standards are required. Emerging frameworks emphasize external validation, avoidance of overfitting, model transparency, interpretability, and harmonized reporting [140,141,142,151,152].

Simultaneously, guidelines increasingly stress the need for population diversity and equity in biomarker evaluation. Many historical validation studies overrepresent individuals of European ancestry or higher socioeconomic status, which may compromise model calibration and amplify disparities when biomarkers are used in primary care [9,147,153].

## 7. Communication, Clinical Stewardship, and GP Responses to Biomarker Demand

### 7.1. Communicating Uncertainty and Limitations

Primary care clinicians often face challenges in explaining complex predictive biomarker evidence to patients in preventive and commercial testing contexts. Effective communication is crucial for making informed decisions and managing expectations. Patients value clarity, honesty about uncertainty, and opportunities to ask questions; however, communication practices regarding biomarker testing often fall short [2,154]. Studies have shown that patients struggle with technical terminology and probabilistic information. Clinicians should use plain language to describe biomarker functions, evidence, and limitations [2,155]. Many biomarkers indicate an increased risk rather than deterministic predictions, requiring explicit emphasis. Addressing uncertainty is essential, whether it is due to assay variability, lack of standardization, limited validation, or evolving science [7,154]. Explaining these limitations prevents over-interpretation and supports realistic expectations.

### 7.2. Shared Decision-Making and Expectation Management

Patients should understand that biomarkers are one component of a broader clinical picture, along with traditional risk factors, symptoms, and family history [2,7]. Clinicians should explain that the results may change as biomarker science evolves, particularly in genomics or immunotherapy [155]. Shared decision-making is crucial when discussing biomarker results. Patient questions, concerns, and preferences improve understanding and engagement [2,154]. Clear dialogue helps patients recognize when acting on the results may not improve outcomes. Written materials, such as brochures and visual aids, improve patient recall and understanding [2]. These tools help patients review information and discuss it with caregivers. Test costs, insurance coverage, and follow-up implications should be addressed proactively [2] to prevent misunderstandings when the results are ambiguous.

### 7.3. Managing Private Biomarker Requests

The rise of private biomarker testing, from genomic risk panels to multi-analyte “wellness packages,” has shifted preventive medicine into commercial territory. GPs now encounter patients seeking interpretation of private biomarkers or requesting tests outside standard indications. These encounters place GPs at the intersection of patient expectations, evidence-based care and resource stewardship.

#### 7.3.1. Educating Patients and Setting Realistic Expectations

Patients often approach biomarker testing with high expectations based on online marketing, anecdotal reports, or misunderstandings about its utility. GPs play a central role in clarifying what biomarker tests can achieve. Evidence shows that clear explanations help patients understand the limitations of unvalidated tests, including false positives, inconclusive results, and over-diagnosis [13,14]. Written summaries or educational materials can support comprehension and counterbalance commercial messages [13]. These conversations create opportunities to reinforce evidence-based prevention strategies, such as lifestyle interventions and standard screening, rather than speculative biomarker-driven insights.

#### 7.3.2. Upholding Evidence-Based Practice and Discouraging Non-Recommended Testing

GPs must serve as gatekeepers of evidence-based care, guiding patients toward biomarker tests with proven validity and discouraging unnecessary private testing. The guidelines state that testing should only be offered when the results inform the outcomes [7]. When patients request biomarker panels outside indications, GPs should explain the potential harms, confusion, or inappropriate follow-up [12,13]. Responding requires transparent reasoning within the context of the patient’s risk profile and health goals, supporting shared decision-making.

### 7.4. When to Refer and When to Decline Testing

Collaboration with specialists is recommended when patients pursue private biomarker testing against GP advice or when the results are complex. This includes genetic counseling for hereditary markers, oncology or endocrinology input for cancer biomarkers, and laboratory expertise for assay interpretation [13,156]. GPs must integrate biomarker findings into the clinical context, avoiding a narrow focus on isolated results. Evidence shows that patients find this integrative approach essential for informed decision-making [7,13].

**Figure 3:**
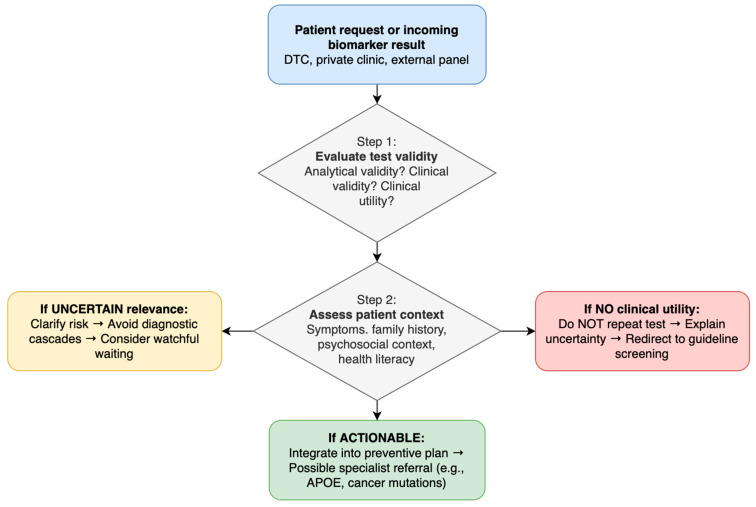
GP workflow for evaluating biomarker requests and incoming test results This flowchart outlines a structured approach for general practitioners when addressing biomarker requests or interpreting results originating from direct-to-consumer (DTC) services, private clinics, or external laboratory panels. It highlights evaluation of test validity, assessment of patient context, and decision pathways for managing non-actionable, uncertain, or actionable findings. Abbreviations: GP, general practitioner; DTC, direct-to-consumer; APOE, apolipoprotein E.

To support clinical decision-making in everyday practice, the key steps in evaluating patient-initiated biomarker requests or externally obtained results are summarized in the workflow shown in Figure 3.

### 7.5. The GP Dekalog for Responsible Biomarker Use in Preventive Practice

As the demand for biomarker testing continues to rise, GPs require clear, actionable principles to guide interpretation, communication, and decision-making in everyday consultations. The following Dekalog summarizes ten core recommendations distilled from the evidence reviewed in this article (Table 1). It is intended as a practical tool to support responsible biomarker use, reduce unnecessary harm, and maintain a patient-centered focus amid expanding commercial and technological pressures.

**Table 1:** Dekalog of responsible biomarker use in preventive general practice.

Number	Principle	Guidance for General Practitioners
1	Prioritize validated biomarkers	Use only biomarkers with strong analytical and clinical validity(e.g., hs-cTnT, hs-cTnI, NT-proBNP, CAC, Lp(a), p-Tau).
2	Do not rely on unvalidated or commercial panels	Avoid DTC tests, SNP-chip rare-variant panels, and unproven “wellness” markers.
3	Use biomarkers only when actionability exists	Order or interpret tests only when results would meaningfully change management.
4	Integrate biomarker results into clinical context	Interpret results alongside symptoms, risk factors, and family history.
5	Communicate uncertainty clearly	Explain probabilistic results, limitations, and lack of deterministic predictions.
6	Avoid diagnostic cascades	Do not repeat low-value tests; avoid unnecessary imaging or referrals.
7	Address psychological impacts	Anticipate anxiety, stigma, or false reassurance; provide balanced counseling.
8	Ensure equity and fairness	Avoid reinforcing disparities created by private testing accessibility.
9	Redirect focus to evidence-based prevention	Emphasize lifestyle interventions, screening programs, and risk-factor management.
10	Uphold stewardship and continuity of care	Use biomarkers judiciously and maintain longitudinal guidance in patient partnerships.

Abbreviations: hs-cTnT, high-sensitivity cardiac troponin T; hs-cTnI, high-sensitivity cardiac troponin I; NT-proBNP, N-terminal pro–B-type natriuretic peptide; Lp(a), lipoprotein(a); CAC, coronary artery calcium; p-Tau, plasma phosphorylated tau; DTC, direct-to-consumer; SNP, single-nucleotide polymorphism.

## 8. Conclusions

The rapid expansion of biomarker-based approaches to disease prevention has created new opportunities and challenges in general practice. This review highlights promising, well-validated biomarkers while noting the substantial pitfalls of widespread test application outside guideline-endorsed indications.

Biomarkers enter GP consultations through patient-driven testing via DTC services and emerging scientific developments that enter the public discourse before clinical guidelines. GPs must balance support for genuine preventive innovations while protecting patients from unnecessary harm and medicalization.

Few biomarkers meet the criteria for analytical validity, clinical validity, and clinical utility for preventive use in asymptomatic adults. Even biomarkers that are strongly associated with disease risk rarely provide actionable insights without targeted interventions. PRS and longevity biomarkers remain immature for routine practice and may increase uncertainty if they are implemented prematurely.

Potential limitations of biomarker testing include the possibility of detecting findings that may not require intervention, creating uncertainty about appropriate follow-up. Some commercial tests provide results without sufficient clinical context, which can leave GPs with the task of clarifying the evidence and outlining realistic implications. Because patients may assign high credibility to biomarker information regardless of its evidentiary basis, clear and proactive communication in primary care remains important.

Within this landscape, the established competencies of general practice—continuity of care, contextualized decision-making, effective risk communication, and responsible use of resources—remain central. GPs are well placed to

• Interpret biomarker findings within the context of patients’ health status, preferences, and daily lives;
• Maintain focus on established preventive strategies;
• Help patients navigate expectations shaped by online or commercial offers;
• Support a measured approach that avoids unnecessary medicalization.

Equity considerations also deserve attention. If access to biomarker testing is largely limited to privately funded pathways, differences in uptake may contribute to disparities in preventive care. A GP-led approach grounded in evidence-based recommendations and accessible testing options can help maintain fairness.

As biomarker technologies continue to advance, some will likely strengthen preventive strategies in primary care. Until clinical utility is clearly established, a careful and patient-centered approach remains appropriate. The ongoing task is to integrate promising innovations in a way that supports, rather than complicates, preventive care.

Ultimately, the role of biomarkers in preventive medicine will be most effective when aligned with the core principles of general practice: proportionality, clarity, shared decision-making, continuity, and trust.

## Data Availability

No new data were created or analyzed in this study. Data sharing is not applicable to this article.

## Abbreviations

The following abbreviations are used in this manuscript:

AD	Alzheimer’s disease
AHA	American Heart Association
APOE	Apolipoprotein E
ASCO	American Society of Clinical Oncology
BRCA1/2	Breast cancer gene ½
CAC	Coronary artery calcium
CAP	College of American Pathologists
DTC	Direct-to consumer
EGTM	European Group on Tumor Markers
ESC	European Society of Cardiology
GAGome	Glycosaminoglycan genome
GP	General practitioner
hs-CRP	High-sensitivity C-reactive protein
hs-cTnI	High-Sensitivity Cardiac Troponin I
hs-cTnT	High-Sensitivity Cardiac Troponin T
HTA	Health technology assessment
LDL-C	Low-density lipoprotein cholesterol
Lp(a)	Lipoprotein(a)
NACB	National Academy of Clinical Biochemistry
NIH	National Institutes of Health
NT-proBNP	N-terminal pro–B-type natriuretic peptide
p-Tau	Plasma p-Tau species
PET	Positron emission tomography
PRS	Polygenic risk score
PSA	Prostate-specific antigen
SANRA	Scale for the Assessment of Narrative Review Articles
